# Adaptive and Compensatory Neural Signatures in Fibromyalgia: An Analysis of Resting-State and Stimulus-Evoked EEG Oscillations

**DOI:** 10.3390/biomedicines12071428

**Published:** 2024-06-27

**Authors:** Lucas Camargo, Kevin Pacheco-Barrios, Lucas M. Marques, Wolnei Caumo, Felipe Fregni

**Affiliations:** 1 Neuromodulation Center and Center for Clinical Research Learning, Spaulding Rehabilitation Hospital and Massachusetts General Hospital, Harvard Medical School, Boston, MA 02115, USA; lcamargo@mgh.harvard.edu (L.C.); kevin.pacheco.barrios@gmail.com (K.P.-B.); 2 Unidad de Investigación para la Generación y Síntesis de Evidencias en Salud, Universidad San Ignacio de Loyola, Lima 15024, Peru; 3 Mental Health Department, Santa Casa de São Paulo School of Medical Sciences, São Paulo 01238-010, Brazil; lucasmurrins@gmail.com; 4 School of Medicine, Universidade Federal do Rio Grande do Sul (UFRGS), Porto Alegre 90010-150, Brazil; wcaumo@hcpa.edu.br; 5 Laboratory of Pain and Neuromodulation, Hospital de Clínicas de Porto Alegre (HCPA), Porto Alegre 90035-903, Brazil

**Keywords:** fibromyalgia, EEG, event-related desynchronization, chronic pain, biomarkers

## Abstract

This study aimed to investigate clinical and physiological predictors of brain oscillatory activity in patients with fibromyalgia (FM), assessing resting-state power, event-related desynchronization (ERD), and event-related synchronization (ERS) during tasks. We performed a cross-sectional analysis, including clinical and neurophysiological data from 78 subjects with FM. Multivariate regression models were built to explore predictors of electroencephalography bands. Our findings show a negative correlation between beta oscillations and pain intensity; fibromyalgia duration is positively associated with increased oscillatory power at low frequencies and in the beta band; ERS oscillations in the theta and alpha bands seem to be correlated with better symptoms of FM; fatigue has a signature in the alpha band—a positive relationship in resting-state and a negative relationship in ERS oscillations. Specific neural signatures lead to potential clusters of neural adaptation, in which beta oscillatory activity in the resting state represents a more adaptive activity when pain levels are low and stimulus-evoked oscillations at lower frequencies are likely brain compensatory mechanisms. These neurophysiological changes may help to understand the impact of long-term chronic pain in the central nervous system and the descending inhibitory system in fibromyalgia subjects.

## 1. Introduction

Fibromyalgia (FM) syndrome is a chronic musculoskeletal condition characterized by generalized chronic pain, allodynia, and hyperalgesia and is frequently associated with sleep disturbances and psychological distress [1]. A previous study estimated the prevalence of FM in the general population to be between 0.2 and 6.6%, and among women this prevalence is between 2.4 and 6.8% [2]. Although researchers have been studying this condition for the past several years, there is no clear biomarker for the diagnosis of FM, and the most common criterion used by clinicians is the presence of chronic pain and other common symptoms, such as fatigue, anxiety, and depression [3]. Also, common interventions to treat FM, such as nonsteroidal anti-inflammatory drugs, antidepressants, anticonvulsants, and opioids, demonstrate limited effects [4]. Therefore, investigation of the physiological mechanisms of FM is pivotal for the understanding of FM mechanisms and the development of novel interventions.

A potential biomarker is offered by electroencephalography (EEG). This assessment is essential in several medical conditions, such as epilepsy, sleep disturbances, and chronic pain [5,6,7]. Oscillatory brain activity can be explored and characterized by specific cognitive functions measured during resting-state, passive, or active responses [8]. The term “brain oscillations” is defined as rhythmic or repetitive electrical activity generated in the central nervous system in response to internal or external stimuli, and they can be recorded by EEG [9]. This technique has been used to investigate several chronic pain conditions, such as fibromyalgia, migraine, and neuropathic pain [6,10,11,12,13]. There is evidence of a relationship between EEG patterns and pain perception; factors such as pain chronicity and emotional state correlate with EEG oscillations, suggesting a connected neurophysiological mechanism of pain and cortical inhibitory drive [10].

Increased theta and beta power are observed in patients with chronic pain during the resting state, which draws attention to thalamocortical dysrhythmia (TCD) in that population [11,12]. In addition to resting-state EEG signals, dynamic states of thalamocortical networks can also be used as potential biomarkers. They are known as event-related desynchronization—related to cortical activation, which reflects a decrease in oscillatory activity due to an internal or external event—and event-related synchronization—related to cortical inhibition, representing increased rhythmic activity. These markers have been explored for specific tasks in clinical trials [13,14,15,16]. In our previous study, we found in the central region that lower alpha and beta power in the resting state and lower ERD in theta and delta bands are associated with higher levels of pain [17]. Thus, using EEG to gain a better understanding of FM brain activity patterns can help to identify some of the mechanisms behind the central sensitization in this condition.

Hence, we aimed to investigate the clinical and physiological predictors of brain oscillatory activity in FM patients, assessing resting-state power, ERD, and ERS during the tasks of movement observation (MO), movement imagery (MI), and movement execution (ME). This study was of an exploratory nature, but based on our previous work, we hypothesized that lower-frequency oscillations at rest and during event-related EEG oscillations would be related to a more compensatory state, and we also expected to find differences in ERS in terms of its relationship with pain control and compensatory mechanisms to identify potential biomarkers of fibromyalgia. 

## 2. Methods

### 2.1. Study Design

We performed a cross-sectional analysis of an ongoing randomized double-blinded clinical trial investigating the combination of transcranial direct current stimulation (tDCS) and aerobic exercise to reduce pain symptoms in patients with fibromyalgia (NCT03371225) [18].

### 2.2. Participants

Inclusion criteria: population between 16–65 years old with a diagnosis of FM pain according to the ACR 2010 criteria, with an average pain level of at least 4 on a 0–10 visual analog scale (VAS) scale for more than six months, without another chronic pain diagnosis; pain resistant to standard medications for chronic pain; subjects must be able to feel the sensation of von Frey fibers on the forearm and be capable of providing informed consent to participate in the study. Exclusion criteria: unstable medical or psychiatric disorder; self-reported history of substance abuse within the past six months; self-reported history of neurological deficits (such as cognitive or motor deficits) or any previous neurosurgical procedure; pregnancy; severe depression (Beck depression inventory (BDI) score higher than 30); current use of opioids in large doses (more than 30 mg of oxycodone/hydrocodone or 7.5 mg of hydromorphone or equivalent); patients excluded if they present increased risk of cardiovascular complications, defined as not fulfilling the American College of Sports Medicine (ACSM) criteria, and, in this case, not cleared by a licensed physician. All patients provided their written informed consent. 

### 2.3. Demographic and Clinical Variables

The self-reported demographic and clinical variables that we collected and included in this study from all the subjects were the following: age; gender; race; fibromyalgia duration (year); visual analog scales (VASs) for pain [19], anxiety [20], depression and sleepiness [21]; quality of life (QoL) scale [22]; Beck depression inventory (BDI) [23]; Pittsburgh sleep quality index (PSQI) [24]; Patient-Reported Outcomes Measurement Information System (PROMIS) for anxiety, fatigue, and pain interference [25]; brief pain inventory (BPI) [26] for pain symptoms and interference subscale; and the revised fibromyalgia impact questionnaire (FIQR) [27].

### 2.4. Conditioned Pain Modulation (CPM)

As in our previous study [17], the protocol involved two conditions, the test stimulus and the conditioned stimulus suggested by Granot et al., 2008 [28] and Nir et al., 2011 [29]. The first step was to determine the pain-60 test temperature (a pain experience score of 60 on a 60–100 NPS) by applying a Peltier thermode (Medoc Advanced Medical Systems, Ramat Yishai, Israel) on the right forearm of the subjects and providing 7 s of three different heat stimuli (43, 44, and 45 °C). Participants rated their pain intensity using a numerical pain scale (NPS) ranging from 0 (‘‘no pain”) to 100 (‘‘the worst pain imaginable”). If the first temperature of 43 °C was considered more than 60, we stopped and provided stimuli at lower temperatures of 41 and 42 °C. When, with the previous three temperatures (43, 44, and 45 °C), the subject did not report pain-60, we applied stimuli at 46, 47, and 48 °C until a pain level of 60 was reached. (If it was not possible to reach pain-60 after applying a temperature of 48 °C, we considered pain-60 as 48 °C.)

Once we had established the pain-60 temperature, we started the test stimulus, applying it for 30 s at that temperature, and the subjects rated their pain intensity three times (10, 20, and 30 s) after the thermode reached the pain-60 temperature, and we calculated the mean scores. For the conditioned stimulus, after five minutes, the subjects immersed their left hand in a bath of water set at 10 to 12 °C for 30 s. Then, still with the left hand immersed in the water, the same pain-60 temperature was applied on the right forearm of the subjects for 30 s, and the subjects again rated their pain intensity three times after the thermode reached the pain-60 temperature (10, 20 and 30 s), and we calculated the mean scores. Also, we calculated the CPM response as the difference between the average pain ratings for the test stimulus minus the average pain ratings for the conditioned stimulus.

### 2.5. Electroencephalography (EEG) Assessment

EEG was recorded using a 64-channel EGI system (EGI, Eugene, OR, USA) describing results from two different periods: (i) a resting-state spectral power EEG period and (ii) blocks of motor execution tasks, as assessed in our previous study [30].

### 2.6. Preprocessing

Following the method applied in our previous studies [30,31], the original data were preprocessed using EEGLab [32] in MATLAB (The MathWorks Inc., Natick, MA, USA 2023) to remove any potential artifacts that could have impaired the analyses. The preprocessing pipeline proposed by Makotow was executed using the Darbeliai EEGLAB plugin. This involved the following steps: (i) bandpasses of 1 Hz (high pass) and 50 Hz (low pass); (ii) downsampling from 1000 Hz to 250 Hz; (iii) re-referencing the channels using the electrode average; (iv) 60 Hz powerline noise correction (frequency in the United States). We eliminated the channels that (i) were flat for longer than three seconds, (ii) showed high-frequency noise greater than two standard deviations, and (iii) showed correlations with neighboring channels less than 0.8 using the Clean_rawdata EEGLAB plugin (v2.2). We performed a visual inspection and rejection of periods containing artifacts prior to performing an independent component analysis (ICA), which was also confirmed by a neurophysiologist. The remaining channels were once more fed into the Infomax ICA calculation using the Darbeliai plugin. This method is effective in identifying artifacts (36, 37) and is significantly related to other techniques. Using the ICLabel toolbox, components associated with heart rate, muscle noise, blinking, and eye movement were eliminated [33].

### 2.7. Resting-State EEG Protocol

To learn the mechanism of brain oscillations during the resting state, EEGs were recorded for 5 min with eyes open, without any cognitive or motor task.

### 2.8. Resting-State Spectral Power Analysis

As described previously regarding the preprocessing steps, we processed the artifact-free data using the pop_spectopo EEGLab function with fast Fourier transformation with 2 s windows with 50% overlap. We calculated the relative power (power in a specific frequency range/total power from 1 to 90 Hz) for the following oscillations: delta (1–3.9 Hz), theta (4–7.9 Hz), alpha (8–12.9 Hz), beta (13–29.9 Hz), and gamma (30–90 Hz), as well as sub-oscillations for low alpha (8–9.9 Hz), high alpha (10–12.9 Hz), low beta (13–19.9 Hz), and high beta (20–29.9 Hz). Considering the objective of the work to explore motor and functional clinical predictors of brain oscillations, the frontal, central, and parietal regions of interest (ROIs) were used to calculate each EEG-related measurement. The selected and averaged electrodes that represent these locations are displayed in Figure S1.

### 2.9. Motor Task Spectral Power Analysis

Similar to our previous research [34], our EEG experimental task was created based on that of Jen-Ren Duann and Jin-Chern Chiou (2016) [35], which compares the observation of a movement (passive and active observation) with the execution of the same movement using the imagination. In detail, the trial’s sequence is as follows: fixation mark before the task condition (−1000 to 0 ms), the task condition (0 to 3000 ms), and a rest post-task and a wait for the next trial (3000 ms to 7000 ms). Combined, the 60 trials occurred randomly, 20 trials for each experimental condition. The three conditions performed and the respective instructions were (i) “clench hand” as the motor task (no video), (ii) watching a video with a hand clenched as the observational task, and (iii) “imagine clenching a hand” as the imagery task (no video). Afterwards, using the pop_spectopo function and fast Fourier transformation (FFT), spectral analysis was performed on the preprocessed data to determine the following standard bands: delta (1–3.9 Hz), theta (4–7.9 Hz), alpha (8–12.9 Hz), low alpha (8–9.9 Hz), high alpha (10–12.9 Hz), beta (13–29.9 Hz), low beta (13–19.9 Hz), high beta (20–29.9 Hz), and gamma (30–90 Hz). Since the right hand was used for all the tasks, these frequencies were mainly assessed for the E20 and E50 electrodes.

### 2.10. Event-Related Spectral Perturbation (ERSP)

Initially, we computed event-related spectral perturbations (ERSPs) to characterize the spectral variations between multiple frequency bands. The amplitude of a frequency component was continuously measured using a three-cycle wavelet Hanning-tapered window [36,37] to create time–frequency plots relative to experimental conditions at each wavelet frequency and time point. Following that, we established two interest windows: event-related desynchronization (ERD)—from 0 ms to 3000 ms—and event-related synchronization (ERS)—from 3000 ms to 7000 ms. Then, to calculate the percentage, the values of each of these time windows for each of the selected bands were divided by the absolute power observed at baseline (time window from −1000 ms to 0 ms), which can be expressed by the following formula: ratio = [absolute power for ERD or ERS window/absolute power at baseline] × 100. Therefore, the ERD and ERS power values for these bands, corrected by the respective baselines, characterized the corresponding results.

### 2.11. Statistical Analysis

We provide the descriptive statistics of the clinical and demographic variables of the study population using means (or percentages) and standard deviations. STATA^®^ 17.0 was used for all statistical analyses. The clinical and demographic characteristics that were previously reported were considered independent variables, and the EEG variables were seen as dependent variables. It is important to note, considering our study design, that our regression analysis was not meant to predict the impact of the independent variables on EEG power values but to be used as a test for associations between the dependent and independent variables. Hence, our findings can be interpreted as statistical tests of correlation, where our most important variables, the EEG variables, were initially tested for associations with clinical and demographic variables.

Prior to determining the values of the unadjusted β coefficients and their 95% confidence intervals (CIs), we conducted a linear univariate analysis to identify which independent variables had a significant association with EEG-dependent variables. Then, following a careful selection of variables (age, biological sex, and fibromyalgia duration), we generated the models for the linear multivariate analyses, where we avoided including potential confounding factors that did not meet the significance level of 0.05 by combining theoretical relevance, confounding assessment (according to the literature, changes of more than 10% in the β coefficients), and statistical criteria (variables that had a *p*-value < 0.2 in the univariate analyses). Variables that failed to maintain statistical significance were eliminated from the model. The four assumptions—linearity, homoscedasticity, independence, and normalcy—defended by Osborne and Waters (2002) [38] were evaluated in order to ensure the statistical quality of the models provided here.

## 3. Results

### 3.1. Demographic and Clinical Characteristics

We included seventy-eight subjects with FM in this study. The mean age was approximately 48 years old, the subjects were predominantly women (88.46%), and most of the subjects self-reported as White (73%). A summary of the clinical and demographic data of our sample is displayed in Table 1.

### 3.2. Neurophysiological Findings

**Figure 1:**
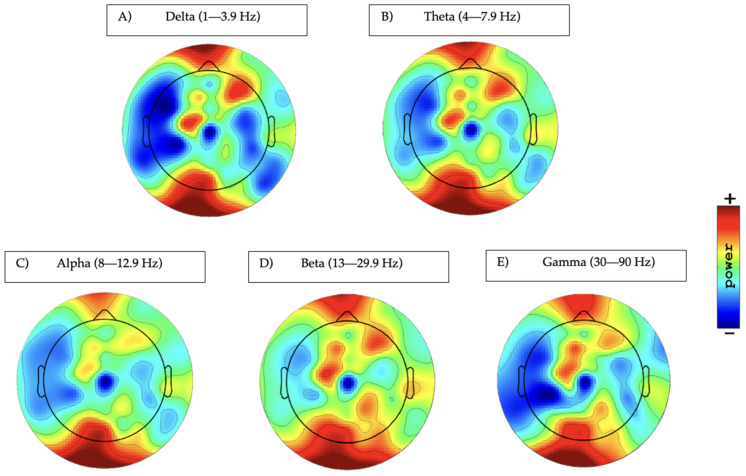
Topographic distribution of scalp plots in resting-state EEG. (**A**): delta power; (**B**): theta power; (**C**): alpha power (range: 35 to 44 dB) (10 × log10 P); (**D**): beta power (range: 23 to 29 dB) (10 × log10 P); (**E**): gamma power (range: −3 to 2.3 dB) (10 × log10 P).

Five subjects were excluded from the EEG analysis due to unavailable data, leaving a total sample of 73 fibromyalgia patients. Descriptive resting-state EEG power analysis for all band and sub-band data in the frontal, central, and parietal regions can be found in Table 2. Also, the topographic distribution of scalp plots in resting-state EEG is displayed in Figure 1.

### 3.3. Time–Frequency Power Analysis

**Figure 2:**
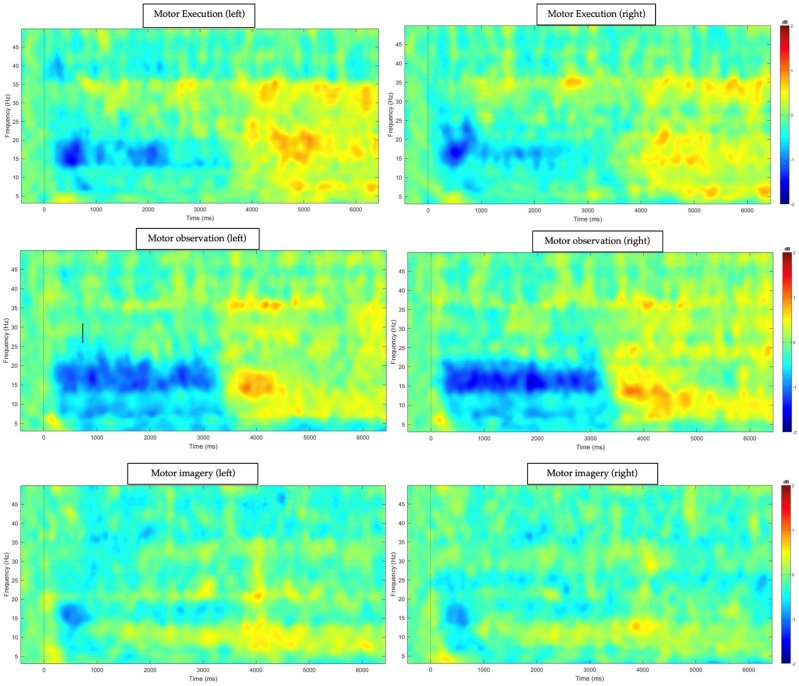
Event-related spectral perturbation (ERSP) for fibromyalgia patients under three conditions. Axes: EEG frequency bands ranging from 1 to 50 Hz. Experimental time window from 1000 ms to 7000 ms. Colors represent absolute power (−2 to 2 dB).

ERD and ERS were calculated using three windows related to our three tasks, corrected by baseline status. The descriptive analyses of ERD and ERS variables are provided in Table 3 and Table 4, respectively. Additionally, time–frequency plots of our motor, observational, and imagery tasks for motor areas (C3 and C4 channels) are presented in Figure 2.

### 3.4. Predictors of Resting-State EEG

In patients with FMS, at baseline, alpha oscillations, in the central area, were associated with an increase in the PROMIS fatigue score (β = 0.029, *p* = 0.013), and these oscillations were associated with a decrease in PSQI (β = −0.016, *p* = 0.035), adjusted by age, biological sex, and the duration of fibromyalgia symptoms. While a higher baseline of beta oscillations in the frontal and parietal areas was associated with a decrease in BPI pain score (β = −0.123, *p* = 0.04; β = −0.10, *p* = 0.04, respectively), in the central area, there was a negative relationship with PROMIS fatigue area (β = −0.011, *p* = 0.028) (model details in Table 5). No other demographic or clinical variables were detected as confounders. Furthermore, regarding theta oscillations at baseline, we found a positive association in the parietal area with pain-60 (β = 0.112, *p* = 0.041), but after the model was adjusted for the same variables, it did not demonstrate statistical significance (β = 0.012, *p* = 0.054), and thus it was excluded from our model. We report the model for resting-state spectral power in Table 5.

### 3.5. Predictors of Event-Related EEG

We conducted multivariate analyses in different areas. For the oscillatory band delta, we found that ERS was associated positively with duration of FM (β = 1.81, *p* = 0.020) in the frontal area, while in the central area, delta ERS had a positive correlation with PROMIS fatigue and duration of FMS and a negative correlation with MEP (β = 5.83, *p* < 0.000; β = 147, *p* = 0.020; β = −25.28, *p* = 0.028, respectively). For the theta band, in the frontal area, ERS was related to a decrease in BPI interference and an increase in duration of FMS (β = −7.32, *p* = 0.033; β = 2.70, *p* = 0.006, respectively), while in the central area, theta ERS only had a relationship with duration of FMS (β = 1.85, *p* = 0.003). For the alpha band, in the frontal and central areas, a greater ERS was associated with a decrease in TSPS and PROMIS (β = 13.97, *p* = 0.005; β = −6.09, *p* = 0.031; β = −12.59, *p* = 0.001; β = −8.02, *p* < 0.000, respectively). For the beta oscillations, a higher beta central ERS at baseline was associated with a decrease in PSQI and an increase in duration of FMS (β = −3.01, *p* = 0.011; β = 1.21, *p* = 0.033, respectively). Finally, gamma frontal ERS and gamma central ERS were positively associated with an increase in BDI (β = 0.81, *p* = 0.008; β = 0.81, *p* = 0.047, respectively). All the models related to ERS were adjusted by age and gender (see Table 6). No significant models related to ERD were found. Due to missing data, we could only include 68 of our subjects in our analysis.

## 4. Discussion

This work sought to understand the pattern of brain oscillation in patients with FM, considering clinical and physiological predictors. The results of our study support that EEG oscillation bands may provide interesting insights for understanding symptom severity and physiological patterns of patients with chronic pain, especially those with FM. In summary, we found (i) a negative correlation between beta oscillations and pain intensity; (ii) that resting-state and stimulus-evoked oscillations show different correlational patterns with chronic pain; (iii) that ERD and ERS also show different correlational patterns, with ERD having no correlation with fibromyalgia clinical characteristics and ERS being highly correlated with several variables; (iv) that fibromyalgia duration is significantly positively associated with increased oscillatory power at low frequencies (delta and theta bands) and also in the beta band; (v) that ERS oscillations in the theta and alpha bands seem to be correlated with better symptoms of fibromyalgia (i.e., less BPI interference and less fatigue and pain summation); (vi) that fatigue symptoms have a specific EEG signature in the alpha band, with a positive relationship with resting-state EEG and a negative relationship with stimulus-evoked oscillatory activity; (vii) and that gamma band ERS is associated with BDI scores.

Regarding the resting state, one of our important findings was the negative correlation between beta oscillations and pain intensity. Beta oscillations seem to be related to cortico-cortical cross-talk and transfer of information. Thus, a decrease in pain with a subsequent increase in beta oscillations may indicate a more physiological state of brain oscillations. It also seems that beta oscillations are important for motor function, especially during active movements (during desynchronization and synchronization). One of the main brain inhibitory neurotransmitters associated with pain processing is gamma-aminobutyric acid (GABA) [39]. Beta oscillations are known to be related to GABAergic activity, produced by inhibitory interneurons in the somatosensory cortices [40,41], and again may be related to this state of increased focus in brain activity so as to inhibit surrounding areas. The presence of long-term chronic pain induces changes in the “pain matrix” structures—the thalamus, the anterior cingulate cortex (ACC), the posterior cingulate cortex (PCC), the insula, the amygdala, the primary and secondary somatosensory cortices, and the periaqueductal gray (PAG)—and, consequently, implicates disruption of the descending modulatory pain system and an imbalance between excitation and inhibition of pain signaling [39,42], and thus may disrupt this potential inhibitory activity of beta oscillations. The presence of high EEG beta oscillations can represent an increase in GABAergic activity, more cortical organization, and a better modulatory effect, which may be a result of reduced pain perception [43]. Our results are in accordance with this theory and demonstrate that high beta oscillations in the frontal and parietal areas are associated with lower pain levels (indicated by lower BPI pain scores) in subjects with fibromyalgia. 

Moreover, several studies on different chronic pain conditions demonstrated this relationship between pain symptoms and beta oscillations. Our previous study showed that lower beta power in the central area was related to higher pain scores in subjects with fibromyalgia as well [17]. Additionally, after observing the EEG variables of 39 patients with spinal cord injury, we could identify the association between higher pain levels and less beta power [44]. Teixeira et al. [43] collected EEG data from 16 patients with chronic peripheral neuropathic pain due to nerve lesions and found reduced GABA levels in pain-related regions and a negative correlation between low beta frequency and VAS pain. The authors suggest that low beta power can be a potential biomarker for chronic neuropathic pain. However, a systematic review performed by Mussigmann et al. [45] found mixed results when analyzing the correlation between pain intensity and beta power. These results reinforce the need for further studies using similar methodologies with chronic pain populations. 

Regarding event-related desynchronization (ERD), we evaluated both the event-related spectral perturbation (ERSP) graphs shown in Figure 2 and the specific relative power requests for the different temporal moments. At later stages of processing (rest post-task period after 3000 ms), it was possible to observe that post-movement beta rebound (PMBR), also known as event-related synchronization (ERS), was strongly associated with error detection and motor inhibition mechanisms, especially in conditions in which the individual needed to inhibit a prepotent response to move the hand when instructed only to watch the video (here, we named this task “motor observation”). Specifically, as presented in the Methods section, we explored the temporal variation in power observed in the different oscillations. However, our results demonstrate, following previous findings of our group [30], that only the variables representing the ERS were significantly associated with clinical characteristics in our sample of fibromyalgia patients. None of the models tested for the different oscillatory bands in the period related to the ERD were significant. This finding was not new and was expected, based on previous studies, since it seems that ERD characteristics are strongly influenced by the preparation for motor execution, which is not expected to be altered in the FMS condition, and, therefore, they are not very sensitive with respect to testing associations with clinical characteristics [30,31]. 

In the literature, it has been reported that ERD can provide information about cortical activation in the sensory–motor (SM) cortex during motor tasks in different neuropsychiatric conditions [46], for instance, (i) Parkinson’s disease seems to delay the onset of SM mu ERD contralateral to the most affected side during voluntary hand contraction, which is even more pronounced in subjects with progressive supranuclear palsy when compared to healthy subjects [47], and (ii) in obsessive–compulsive disorder (OCD), delay activation of mu ERD contralateral to movement was observed, which suggests improper movement preparation, due to a dysfunction of the basal ganglia and the cortical–subcortical system [48]. Additionally, in our previous research, we observed 62 subjects with phantom limb pain (PLP) due to unilateral traumatic lower-limb amputation, and we found motor cortex asymmetry reorganization in this population, assessed by transcranial magnetic stimulation (TMS) and magnetic resonance imaging (MRI), which evidenced less cortical organization in the affected motor cortex (contralateral to the amputation) and a reduction in the volume of the affected hemisphere.

### 4.1. Stimulus Evoked Oscillations: Event-Related Synchronization (ERS)

Event-related synchronization (ERS) is interpreted as active cortical processing and engagement in an activity, while ERD is thought to correspond to a more specific network activation and inhibition of the surrounding areas [49]. We observed that delta, theta, alpha, beta, and gamma oscillations were associated with clinical characteristics in our sample of fibromyalgia patients. 

In our models, we found a positive correlation of frontal and central delta and theta ERS and central beta ERS with fibromyalgia duration, which means that long-term FM symptoms are associated with increased ERS after hand movement in these low-frequency (delta and theta) and beta bands. As discussed before, long-term chronic pain induces changes in the structure and function of the “pain matrix”, which is also related to failure to deactivate regions of the default mode network (DMN) and attentional networks when performing cognitive tasks in this population [50]. As part of brain adaptation, areas correlated with pain perception and long duration, such as the somatosensory cortices, the insula, or the ACC, frequently show more brain activity [51]. Also, higher ERS in beta bands can be associated with long chronic pain due to their relationship to GABAergic activity in modulating the descending inhibitory pain system [43]. The downshift of brain oscillations to low-frequency bands (delta and theta bands) can be explained by compensatory mechanisms for cortical–subcortical dysregulation due to the theory of thalamocortical dysrhythmia, which is frequently associated with several chronic pain conditions [52,53,54]. Das et al. [55] observed the importance of DMN in cognitive tasks and found a high intra-network synchronization phase in low-frequency bands and a stronger cross-network synchronization phase in beta bands, which elucidates the importance of delta, theta, and beta bands for the stability of brain networks. 

In addition, we observed higher ERS in theta bands in frontal areas and in alpha bands in frontal and central areas, which seems to be correlated with fewer symptoms (better TSPS and fatigue scores). Higher theta frontal ERS indicates lower BPI interference levels, and we observed a similar pattern in our previous study on chronic knee osteoarthritis patients [56], which may indicate potentially salutogenic and adaptive oscillations associated with fewer symptoms. 

Interestingly, fatigue symptoms demonstrated a positive association with higher alpha bands in the resting state and a negative association with higher ERS in alpha bands in the frontal and central areas. Alpha oscillations have been known for their role in controlling active inhibition and task-irrelevant and task-relevant facilitation brain regions [57,58]. In the literature, an increase in alpha power is associated with sustained levels of attention and fatigue [59], while increased task-related alpha oscillations seem to reflect a decrease in attention and drowsiness, especially when maintaining driving alertness, as suggested by Zhang et al. [60]. 

Additionally, our findings demonstrated an association between higher gamma ERS (in the frontal and central areas) and higher BDI scores. A correlation between the limbic system and gamma oscillation has already been described in the literature [61,62,63]. Also, the authors argue that the gamma band has a key role in cognitive function and emotional processes [61,63]. According to Li et al. [63], depressed subjects demonstrated more gamma activity and negative emotional words than the control group, and similar findings were also reported by Siegle et al. [64]. Nevertheless, brain network signals in the depressive population described in the literature present different trends.

### 4.2. Limitations

One of the main limitations of this study was the absence of a control group, as all subjects were patients with FM, so we could not compare our results with healthy subjects, and we included only baseline values from our ongoing clinical trial. We assessed motor execution with the right hand without evaluating the differences between both hemispheres, with the two hands executing the same task. Also, motor imagery may have produced less of a response due to its demanding less attention from the patients, and some of them became tired after a few minutes of the trial. Another important limitation is the cross-sectional nature of the study, which is suitable for exploring associations but not for establishing causal relationships. Therefore, future trials should address these limitations by including a control group and bilateral motor tasks and assess short- and long-term neurophysiological changes in order to improve the understanding of EEG signals and clinical aspects in the FM population.

## 5. Conclusions

Even though this study was conducted with fibromyalgia subjects only, we found significant and important neural signatures of several symptoms and characteristics of fibromyalgia, including pain intensity, fatigue, fibromyalgia duration, and depression intensity. This study also opens up the possibility of novel studies investigating laterality changes, given that we only assessed motor execution with the right hand. All the neurophysiological changes we showed here may help our understanding of the impact of long-term chronic pain in the central nervous system and the changes in the descending inhibitory system in fibromyalgia subjects. Resting-state oscillations and ERS seem to provide potential biomarkers for several neuropsychiatric conditions, but the investigation into the fibromyalgia syndrome is still in its infancy. 

## Figures and Tables

**Table 1:** Demographic and clinical characteristics (*n* = 78).

Characteristics	Mean or %	SD
Age	47.94	11.42
Biological sex (female, %)	69 (88.46%)	
Race:		
American Indian or Alaska Native	1 (1.3%)	
Asian	2 (2.6%)	
Black or African American	8 (10%)	
White	57 (73%)	
More than one race	7 (8.9%)	
Unknown or not reported	3 (3.85%)	
Duration of fibromyalgia (years)	11.26	8.71
Pain level (VAS)	5.98	1.83
VAS anxiety	4.32	2.75
VAS depression	3.8	2.9
VAS sleepiness	6.12	2.65
BDI	16.55	8.71
PSQI	12.25	4.39
QoL	68.87	14.58

**Table 2:** Resting-state EEG—relative power (%) N = 73.

Band	Frontal (Mean ± SD)	Central (Mean ± SD)	Parietal (Mean ± SD)
Delta	14.06 (11.77)	12.99 (11)	12.98 (11.62)
Theta	11.45 (7.9)	11.23 (8.08)	10.94 (11.67)
Alpha	62.48 (23.51)	63.21 (23.51)	64.23 (21.56)
Low alpha	26.30 (11.67)	25.49 (9.97)	26.22 (10.96)
High alpha	30.42 (14.86)	31.54 (14.78)	32.22 (14.98)
Beta	10.56 (9.83)	11.18 (10.25)	10.46 (8.64)
Low beta	6.48 (5.07)	7 (5.52)	6.82 (5.13)
High beta	4 (5.27)	4 (5.48)	3.56 (4.11)
Gamma	0.46 (0.90)	0.44 (0.81)	0.43 (0.93)
Low gamma	0.32 (0.50)	0.31 (0.46)	0.29 (0.49)
High Gamma	0.14 (0.49)	0.13 (0.40)	0.14 (0.46)

**Table 3:** Descriptive analysis of event-related desynchronization (ERD) as percentages. N = 73.

Band	Motor Execution (Mean ± SD)	Motor Observation (Mean ± SD)	Motor Imagery (Mean ± SD)
Delta	107.8 (48.7)	102.8 (40.8)	105.7 (55.8)
Theta	106.0 (47.3)	94.9 (33.3)	99.0 (30.2)
Alpha	102.7 (23.0)	93.6 (17.6)	105.6 (28.4)
Beta	99.8 (26.4)	88.7 (19.1)	104.0 (27.9)
Gamma	100.4 (42.1)	93.2 (30.2)	91.0 (27.1)

**Table 4:** Descriptive analysis of event-related synchronization (ERS) as percentages. N = 73.

Band	Motor Execution (Mean ± SD)	Motor Observation (Mean ± SD)	Motor Imagery (Mean ± SD)
Delta	111.8 (48.6)	102.8 (32.4)	105.4 (43.8)
Theta	119.3 (49.7)	107.1 (39.7)	106.3 (43.6)
Alpha	120.1 (51.1)	122.5 (50.7)	98.6 (14.1)
Beta	122.5 (35.7)	114.9 (27.2)	98.8 (17.8)
Gamma	107.2 (34.9)	98.3 (28.6)	95.2 (22.4)

**Table 5:** Predictors of resting-state EEG.

Variables	Unadjusted Effects Coefficient				Adjusted Effects Coefficent *			
	Beta coefficient	95% CI	*p*-value	R^2^	Beta coefficient	95% CI	*p*-value	R^2^
Alpha Central				0.12				0.26
PROMIS fatigue	0.029	0.007 to 0.052	0.013		0.024	0.002 to 0.047	0.035	
PSQI	−0.016	−0.031 to −0.001	0.035		−0.010	−0.025 to 0.005	0.181	
Beta Frontal								0.20
BPI pain	−0.123	−0.023 to 0.002	0.086	0.04	−0.014	−0.0273 to −0.0003	0.046	
Beta Central								0.24
PROMIS fatigue	−0.011	−0.021 to −0.001	0.028	0.24	−0.009	−0.018 to −0.0002	0.046	
Beta Parietal								0.21
BPI pain	−0.10	−0.023 to 0.002	0.10	0.04	−0.012	−0.024 to −0.0001	0.048	

* Adjusted by age, biological sex, and fibromyalgia duration. N = 73.

**Table 6:** ERS multivariate model.

Variable	Beta Coefficient *	95% CI	*p*	R^2^
Delta Frontal ERS				0.17
Duration of FMS	1.81	0.29 to 3.33	0.020	
Delta Central ERS				0.50
PROMIS fatigue	5.82	2.80 to 8.85	<0.000	
MEP	−25.28	−47.70 to −2.85	0.028	
Duration of FMS	1.47	0.23 to 2.70	0.020	
Theta Frontal ERS				0.17
BPI interference	−7.32	−14.0 to −0.60	0.033	
Duration of FMS	2.70	0.82 to 4.57	0.006	
Theta Central ERS				0.17
Duration of FMS	1.85	0.66 to 3.05	0.003	
Alpha Frontal ERS				0.17
TSPS	−13.97	−23.56 to −4.37	0.005	
PROMIS fatigue	−6.09	−11.59 to −0.58	0.031	
Alpha Central ERS				0.35
TSPS	−12.59	−19.83 to −5.35	0.001	
PROMIS fatigue	−8.02	−12.26 to −3.79	<0.000	
Beta Central ERS				0.14
PSQI	−3.01	−5.31 to −0.72	0.011	
Duration of FMS	1.21	0.10 to 2.34	0.033	
Gamma Frontal ERS				0.14
BDI	0.81	0.40 to 2.61	0.008	
Gamma Central ERS				0.076
BDI	0.81	0.01 to 1.61	0.047	

* Adjusted by age and biological sex. N = 68.

## Data Availability

The data that support the findings of this study are available from the corresponding author upon reasonable request.

## Supplementary Materials

The following supporting information can be downloaded at: https://www.mdpi.com/article/10.3390/biomedicines12071428/s1, Figure S1: EGI 64-channel HydroCel Geodesic Sensor Net is displayed below.
