# Supplementary figures and images for: Adaptive and Compensatory Neural Signatures in Fibromyalgia: An Analysis of Resting-State and Stimulus-Evoked EEG Oscillations
Source: Biomedicines. 2024 Jun 27;12(7):1428. doi: 10.3390/biomedicines12071428 (PMC11274211; doi:10.3390/biomedicines12071428)

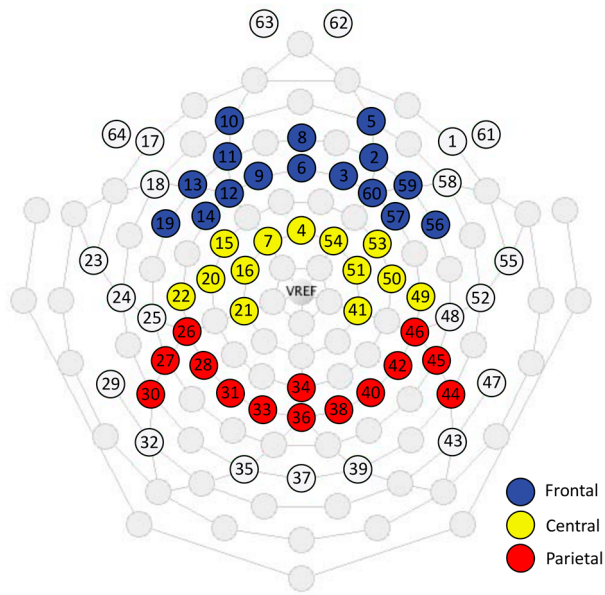

Figure S1. EGI 64-channel HydroCel Geodesic Sensor Net is displayed below.

Supplement: Supplementary file 1 [file biomedicines-12-01428-s001.zip › biomedicines-3008710-supplementary.pdf]
